# A few ethical issues in translational research for gene and cell therapy

**DOI:** 10.1186/s12967-019-02154-5

**Published:** 2019-11-28

**Authors:** Luciana Riva, Carlo Petrini

**Affiliations:** Bioethics Unit, Istituto Superiore di Sanità (Italian National Institute of Health), Via Giano della Bella 34, 00162 Rome, Italy

**Keywords:** Translational research, First in human trials, Cellular and gene therapy, Research ethics

## Abstract

**Background:**

Although translational research for drug development can provide patients with valuable therapeutic resources it is not without risk, especially in the early-phase trials that present the highest degree of uncertainty. With the extraordinary evolution of biomedical technologies, a growing number of innovative products based on human cells and gene therapy are being tested and used as drugs. Their use on humans poses several challenges.

**Methods:**

In this work, we discuss some ethical issues related to gene and cell therapies translational research. We focus on early-phase studies analysing the regulatory approach of Europe and the United States. We report the current recommendations and guidelines of international scientific societies and European and American regulatory authorities.

**Results:**

The peculiarity of human cell- or tissue-based products and gene therapy has required the development of specific regulatory tools that must be continually updated in line with the progress of the research. The ethics of translational research for these products also requires further considerations, particularly with respect to the specificity of the associated risk profiles.

**Conclusions:**

An integrated ethical approach that aims for transparency and regulation of development processes, the support of independent judgment in clinical trials and the elimination of unregulated and uncontrolled grey areas of action are necessary to move gene and cell therapy forward.

## Background

With the rapid advancement of biomedical technologies it is today possible to develop different innovative products to use as drugs, in particular human cell- or tissue-based products and gene therapy. Cell and gene therapies can be generically described as medical procedures in which cells or genes represent the medicinal product. They have very complex characteristics and their use on humans poses several challenges. Over the past 25 years, gene and cell therapy approaches have grown enormously and, especially in the beginning, clinical studies have sometimes resulted in significant adverse events for patients [1]. So-called *living drugs* are unstable from a biological point of view and in most cases are not completely predictable in terms of their effects. The variability depends primarily on manipulative processes and, furthermore, the new environment with which they interact can influence their behaviour, as in the case of cells re-injected into the patient’s body. Decades of research have produced some general information on the mode of action of drugs in the human body, while the mechanisms of action of cellular therapies are still difficult to define. Unlike traditional synthetic products, which are metabolised and expelled from the body, cell-infusion or gene therapy may be irreversible. The peculiarity of these products has required the development of specific regulatory tools that must be continually updated in line with the progress of the research. The ethics of translational research therefore requires further considerations with respect to the specificity of the associated risk profiles.

At the beginning of the century, with the development of nanotechnologies, the potential risk associated with the use of new materials on man was a concern. Since nanomaterials were relatively new substances, understanding and predicting risks were the most significant problems in relation to risk minimisation [2]. The critical issue is unpredictability: new materials have novel properties that may affect humans in unpredictable ways. This is the same problem that has arisen in the last two decades with products derived from tissue engineering and cell manipulation. To date, almost 2600 gene therapy clinical trials have been completed, are ongoing or have been approved worldwide [3]. Recently, in vivo genome editing trials have also been authorized [4]. Aside from the safety risk, human genome editing, in particular germline editing, poses additional ethical questions that are beyond the scope of this work.

Regarding stem cell research, there are over 5000 registered clinical trials on ClinicalTrials.gov and that number is growing every day. Incidentally, it should be noted that a worrying phenomenon of direct-to-consumer interventions has occurred with regard to stem cell therapies. Presumed treatments for the most varied pathologies are sold to patients, who are highly vulnerable, outside the control of the competent authorities. It is important to emphasise that, in order to protect people, no experimentation can take place outside a regulated context and without shared scientific validation [5].

## Methods

We focus on translational research in early-phase studies of gene and cell therapy first analysing its general meaning and the related ethical considerations. Than we examine the regulatory approach of Europe and the United States and the current guidelines and recommendations of the European Medicine Agency (EMA) and the Food and Drug Administration (FDA) respectively. We also report the positions of international scientific societies that addressed, in particular, the criteria for a correct and timely translation of stem cell research to clinics.

### Translational research in early-phase studies of gene and cell therapies

Translational research can be defined in many different ways. Overall, it encompasses activities from the laboratory bench to clinical practice, and including health policy actions. Ethical problems arise at each of these levels of research and translation of the knowledge acquired. Some are common to every process of knowledge transfer: the identification of the principles and values that should guide the setting of priorities; the choice of the types of results that should be considered; the identification of the responsibilities of the various stakeholders (researchers, research founders, policy-makers, decision-makers, etc.); the identification of the mechanisms that can be adopted for ethical supervision and the identification of processes that should be subject to such supervision. Other problems are specific for each level of translational research [6, 7]. Early-phase studies, in particular “first-in-human” (FIH) studies, arise a series of ethical questions that are more pronounced in the case of cellular and gene therapy products.

Key ethical issues in FIH studies can be identified as follows: difficulty in evaluating preclinical research; difficulty in assessing the risk-to-benefit ratio; conceptualisation and estimation of patient benefits and/or social benefits; application of the principle of justice; criteria for inclusion/exclusion of participants; process of information and consent; and risk of therapeutic misconception (Table 1).

**Table 1:** Overview of key ethical challenges of CGT (cellular and gene therapy) early-phase clinical trials [13–17]

Overview of key ethical challenges of CGT early-phase clinical trials
***Key ethical challenges of early-phase clinical trials***
Difficulty in evaluating preclinical research
Difficulty in assessing the risk-to-benefit ratio
Conceptualisation and estimation of patients benefits and/or social benefits
Criteria for inclusion/exclusion of participants
Process of information and consent
Risk of therapeutic misconception
***Some distinctive features of CGT products that have an influence on the design of early-phase clinical trials***
Potential for prolonged biological activity after a single administration
High potential for immunogenicity
Need for relatively invasive procedures to administer the product
Preclinical data not informative as for small molecule pharmaceuticals
Unique complexities of CT products due to the dynamic nature of living cells (stem cells may undergo transformation and begin forming tumours)
In GT products, expression of a delivered gene may be uncontrolled and may interfere with normal function of a critical enzyme, hormone or biological process in the recipient
Genomic alteration in the recipient could cause activation or inactivation of neighbouring genes and generate benign or malignant tumours

Starting from the main international ethical codes and declarations on clinical research, there is a consensus that basic laboratory and animal research must precede clinical research in order to develop safe and effective therapies and medical procedures. The Nuremberg Code states that “The experiment should be so designed and based on the results of animal experimentation and knowledge of the natural history of the disease or other problem under study that the anticipated results will justify the performance of the experiment” (Nuremberg Military Tribunals, 1948–1953). The Declaration of Helsinki also states a similar requirement (WMA, 1964–2013, article 18) and provides that: “Every medical research study involving human subjects must be preceded by careful assessment of predictable risks and burdens to the individuals and communities involved in the research in comparison with foreseeable benefits to them and to other individuals or communities affected by the condition under investigation”. When healthy subjects are recruited for first-in-human trials, it is reasonable to require that the relevant preclinical evidence concerning possible risks be even more robust than when patients with serious underlying pathologies are involved [6]. Overall, for decision-makers, the evaluation of preclinical evidence represents a judgment that is by no means simple [8, 9]. When, in 2016, the first-in-human phase 1 CRISPR gene editing cancer trials (ex vivo) began to be proposed in the United States, some authors wondered if the translation time was premature, that is not justified by the validity of preclinical evidence. If so, the subjects would be put at risk for no potential benefit, a “leap of faith” that cannot be justified either by claims of urgent medical need [10]. The complexity of establishing the scientific validity of preclinical studies supporting the transition to a phase 1 clinical trial for the CRISPR genomic editing methodology, as well as for gene therapy in general, clearly has its own specificity with respect to traditional drugs.

Experimental studies by their nature are characterised by some degree of uncertainty about the results and possible risks for the participants, especially in the initial phases of human research. All international documents and guidelines require that an appropriate risk/benefit ratio should be the basis of a clinical trial. The fundamental purpose of such a requirement is to prevent the research subjects being exploited or harmed. As has been clearly underlined in the Belmont Report, there are no quantitative techniques or mathematical formulae to measure the risks entailed by research procedures. Nevertheless, the process of weighing risks and benefits should be non-arbitrary as far as possible [11]. In approaching risk overall, there is agreement that three conditions must be met: (i) the potential risks to individual subjects must be minimised; (ii) the potential benefits to individual subjects must be enhanced; and (iii) the potential benefits to individual subjects and society must be proportionate to or outweigh the risks [12]. Investigators and Institutional Review Boards (IRB) should also systematically assess the nature of risk and benefits, although researchers and members of IRBs may also have divergent views on the risks arising from a treatment. The conceptualisation of benefits itself requires decision-making and value choices. It is necessary to establish what constitutes a therapeutic benefit for a patient and, if there are no direct benefits, risk to the trial participants must be balanced against potential benefits to society. The likelihood of therapeutic benefits for patients in FIH trials is generally very slight and there is therefore a need to carefully evaluate what constitutes compelling societal benefits [13].

In 2018, The International Society for Stem Cell Research (ISSCR) published the document: “Stem Cell-Based Clinical Trials: Practical Advice for Physicians and Ethics/Institutional Review Boards”, a guide designed to support physicians and ethics/institutional review in evaluating early-phase, stem cell-based clinical trials. The document emphasizes how not everyone involved could be versed in assessing the merits of cell-based trials, especially clinicians and local institutional review boards who could therefore relying heavily on the information given to them by the sponsors of the trial [14].

Fair subject selection is a key dimension in making clinical research ethical. Generally, there is disagreement over which types of subjects are appropriate to recruit for FIH trials and whether it is right to recruit healthy subjects [13]. Many of the FIH studies involving cells and gene-transfer agents are planned for seriously ill patients who have exhausted the therapeutic possibilities. It is important to consider that even in these cases, not all risks are ethically justifiable by the absence of alternatives and that the patient must be as aware as possible of the significance and of the uncertain nature of the treatment. The recent history of the development and commercialization of chimeric antigen receptor T cell therapies (CAR-T cell therapies) shows some relevant ethical issues. These personalized gene therapies against cancer act through the genetic engineering of the patient’s T lymphocytes. Recently, some of these products have gained market access in Europe and United States despite evidence of serious side effects [15]. Overall, despite enthusiasm for positive results, there is still a great deal of uncertainty regarding the long-term benefits and risks of even approved CAR-T therapies. Safety concerns and side effects are part of clinical research but the personalized nature of these therapies places them outside the traditional paradigms of risk–benefit assessment. Professionals should carefully evaluate suitability for treatment on a case-by-case basis and promote ethical recruitment into clinical trials. Unlike already approved CAR-T products, whose use is limited to last-resort patients, clinical trials are starting to evaluate CAR-T cell therapies as first or second line treatments options. That is also in the eventuality that the patient has available a series of therapeutic alternatives [16]. The speed of such a movement towards a wider market should be carefully modulated and take place in strict adherence to ethical principles and the protections of patient’s rights and interests.

It is an undisputed principle in biomedical research involving human beings that, prior to the start of the trial, participants must be helped to understand the uncertainty, risk of adverse events, and any therapeutic benefit in order to express meaningful informed consent. Therapeutic misconception is a phenomenon documented by numerous studies and indicates that the patient might confuse scientific research with therapeutic treatment, presumably because he/she overestimates the benefits [17]. This situation is particularly likely to occur in the case of patients who are highly vulnerable and have exhausted the therapeutic possibilities.

Stem cell-based approaches are beginning to be tested in clinical trials on neurodegenerative disorders. These could also include first-in-human intracerebral transplantation of cells derived from human embryonic stem cells (hESCs) and inducible pluripotent cells (iPSCs) [18].

As has been emphasized by some authors, the uncertainty and risks involved in all early clinical trials are increased when, for instance, a pluripotent stem-cell-based therapeutic is been tested and the target is the brain because any side effects have the possibility of affecting the patient’s cognitive functions [19].

Furthermore, in cases of patients with cognitive impairment, it may be more difficult to obtain a valid informed consent.

### Regulatory approach

Europe and the United States have different legal and regulatory regimes for approving gene and cell therapies. However, the changes and the evolution of these innovative therapies have represented a challenge for both supervisory systems, which must continually adapt [20] in an ongoing dialogue with all stakeholders. In the United States, biological drug products are subject to US Food and Drug Administration (FDA) pre-market approval. These include cellular therapy products, human gene therapy products and certain devices related to cell and gene therapy.

In 2015, the FDA’s Center for Biologics Evaluation and Research (CBER), which regulates specifically cellular therapy and human gene therapy products, prepared guidance to assist sponsors and investigators in designing early-phase clinical trials for cellular therapy (CT) and gene therapy (GT) products (collectively CGT products). This guidance contains recommendations regarding clinical trials in which the primary objectives are initial assessments of safety, tolerability or feasibility of administration of the investigational products. The document focuses on those aspects of early-phase clinical trial design that are different for CGT products compared to other types of products (Table 1) and stresses the need for a case-by-case approach for the design of each clinical trial [21]. In 2018, moreover, the CBER released specific gene-therapy guidelines for classes of diseases such as rare disease and haemophilia [22, 23].

In Europe, advanced therapy medicinal products (ATMPs) are medicines for human use based on genes, tissues or cells, regulated by the Regulation (EC) No 1394/2007 for which marketing is authorised centrally via the European Medicines Agency (EMA). Specifically, these are gene-therapy, somatic-cell-therapy and tissue-engineered products. Stem cell-based therapies are classified as ATMP when the cells have undergone “substantial manipulation” or are used for a function different from that which they originally exerted in the organism [24]. In 2011, the European Medicines Agency (EMA) drew up a reflection paper on stem cell-based medicinal products to stress the fact that considerable attention must be paid to the development of these medicines and to the overall translational research approach [25].

European Medicines Agency developed guidelines to help producers prepare Marketing Authorisation Applications for human medicines. It has developed numerous guidelines for gene-therapy medicinal products and for cell-therapy and tissue-engineering products, and specific guidance regarding clinical trial application for advanced-therapy investigational medicinal products (ATIMPs). As reported in the document “Guideline on quality, non-clinical and clinical requirements for investigational advanced therapy medicinal products in clinical trials” [26], the clinical development of ATIMPs applies the same principles as for other IMPs, according to Annex I to Regulation (EU) No 536/2014 [27]. However, the distinctive characteristics of these products could have an impact on the trial design, specifically for early-phase trials. Distinctive features of ATMPs include complexity of products, limitations on extrapolating relevant information such as immunogenicity, on- and off-target effects and tumourigenicity from animal data, and uncertainty about frequency, duration and nature of side effects [26]. The guideline specifies that, according to Directive 2001/20/EC and Regulation (EU) No 536/2014, an evaluation of the anticipated benefit and risk should be included in the trial protocol: sponsors have a duty to define the benefit-risk assessment and specify just how potential risks will be addressed and minimised. In addition, the rationale and justification for the choice of the study population should be explained. The document also focuses on first-in-human (FIH) studies, defined as a subset of exploratory studies, when the ATIMP is translated for the first time from non-clinical studies to humans, stressing that trial phases in ATMP development are usually not as clear-cut as they might be for other product types. Actually, the development of these products is likely to change the stage-structure of clinical trials as we understand it today for traditional drugs. For ATMPs, the collection of data within the concept of the risk-based approach is an on-going process prior to the submission of a Marketing Authorisation Application (MAA) [26]. In early-phase trials, single-arm studies are frequently used instead of Randomised Controlled Trials (RCTs), resulting in significant heterogeneity across the total evidence landscape. As some authors have underlined, it is necessary to shape new approaches for the analysis of clinical evidence, which is still very limited for cell and gene therapies [28].

In 2017, the Lancet Commission on Stem Cells and Regenerative Medicine reported that the previous 10 years had seen exponential growth in experimental therapies, broadly defined as regenerative medicine (very generic definition that includes cell and gene therapy, tissue engineering, and new generation drugs), with a relatively small number of clinical successes and an enormous burden of expectation [29]. The Commission reports that existing research in the field is hampered by the frequent absence of strong preclinical evidence, poor trial design, and poor and inconsistent reporting, particularly of non-randomised trials. In the document, the authors provide a series of recommendations, including the creation of an international register of cell and biological experimental interventions, possibly within the European Medicines Agency and US Food and Drug Administration, with a careful process of review to guarantee the scientific soundness of trials. In particular, they focus on the risk profile for cell therapies, emphasising that uncontrolled stem cell therapies have a particularly problematic risk structure and informed consent struggles to adequately protect individual interests outside a strong governance framework. Clearly, when the information available about risks and benefits is uncertain, it will be difficult, if not impossible, for individuals to control their risks through informed consent alone.

## Results

Regulatory certainty and institutional control are essential to avoid the establishment of grey areas of action in which patients are at risk of exploitation. The famous “Stamina case” in Italy is an example of this. Patients opposed the State for access to an alleged innovative stem cell therapy called “Stamina” based on the use of mesenchymal stem cells (MSC) and intended for the treatment of neurodegenerative diseases. In the context of a highly controversial legal battle, these manipulated cells were administered to numerous patients in public hospitals, without a standardized study protocol upstream [5].

Furthermore, specific professional communities must also promote professional standards that lead to more ethical conduct because even physicians themselves may be at risk of serious bias or conflict of interest [30]. It is also very important to consider this aspect with regard to certain experimental uses of these products that fall under the responsibility of the clinician, such as the so-called “hospital exemption”.

In 2016, the International Society of Stem Cell Research (ISSCR) developed specific guidelines for the field of stem cell research to encourage the correct and timely translation of stem cell research to clinical environments [31]. The documents open with the statement: “the primary societal mission of basic biomedical research and its clinical translation is to alleviate and prevent human suffering caused by illness and injury. All such biomedical research is a collective effort”. Some of the recommendations may apply for any basic research and clinical translation efforts, while others respond to challenges specific of stem cell-based research: for example, the vulnerability and pressing medical needs of patients with serious illnesses that currently lack effective treatments, and public expectations about medical advance and access. With regard to approaches intended to ensure that informed consent is valid in early-phase trials of stem cell interventions, the document suggests: (a) conducting informed consent discussions that include an individual independent from the research team; (b) explaining to prospective subjects that major therapeutic benefits in early-phase studies are exceedingly rare; (c) testing prospective subjects on comprehension before accepting their consent; (d) requiring a “cooling-off” period between provision of consent discussions and acceptance of consent; (e) avoiding language that has therapeutic connotations, for example, using words like agent or cells rather than therapy; and (f) supplementing consent forms with additional educational materials. As appropriately specified, both legislation and guidelines of this type should be periodically revised to adapt to scientific progress and changes in social priorities.

## Conclusions

In the drug development process, FIH trials involve the greatest degree of uncertainty and raise a number of ethical issues, with the most important point at issue being the need to provide adequate protection for those involved. Compared to traditional drugs, pharmacological treatments based on human cell and gene therapy have much more complex characteristics and mechanisms of action that are often difficult to understand and predict. Independent judgement and oversight from IRBs are essential requirements irrespective of whether these product are being used in a clinical trial or in other types of situations such as compassionate use or under the so-called “hospital exemption”. At present, it is important to make sure that these Boards have the necessary competencies for assessments of this type. Professional communities should also promote the adoption of standards that lead to ethical conduct, because even physicians themselves might be at risk of serious bias or conflict of interest. An integrated ethical approach that aims for transparency and regulation of development processes, the support of independent judgment and the elimination of unregulated and uncontrolled grey areas of action are necessary to move gene and cell therapy forward.

## Data Availability

Not applicable.

## Abbreviations

ATMPs: advanced-therapy medicinal products
CAR-T: chimeric antigen receptor T cell
CGT: cellular and gene therapy
CRISPR: clustered regularly interspaced short palindromic repeats
EMA: European Medicines Agency
FDA: Food and Drug Administration
FIH: first-in-human
hESC: human embryonic stem cells
IRB: Institutional Review Board
ISSCR: International Society of Stem Cell Research
IND: Investigational New Drug
iPSCs: induced pluripotent stem cells
MAA: Marketing Authorisation Application
MSC: mesenchymal stem cells
RCTs: Randomised Controlled Trials
